# The prevalence of pilus islets in *Streptococcus pneumoniae* isolates from healthy children in Indonesia

**DOI:** 10.1099/acmi.0.000184

**Published:** 2020-12-03

**Authors:** Dodi Safari, Feby Valentiya, Korrie Salsabila, Wisiva Tofriska Paramaiswari, Wisnu Tafroji, Sven Hammerschmidt, Sri Rezeki Hadinegoro

**Affiliations:** ^1^​ Eijkman Institute for Molecular Biology, Jakarta, Indonesia; ^2^​ Department of Biochemistry, Faculty of Mathematics and Natural Sciences, Bogor Agricultural University, Bogor, Indonesia; ^3^​ Department of Molecular Genetics and Infection Biology, Interfaculty Institute for Genetics and Functional Genomics, Center for Functional Genomcis of Microbes, Universität Greifswald, Greifswald, Germany; ^4^​ Department of Child Health, Faculty of Medicine, University of Indonesia/Dr Cipto Mangunkusumo Hospital, Jakarta, Indonesia

**Keywords:** *Streptococcus pneumoniae*, pilus islet, Lombok Island, Indonesia

## Abstract

*
Streptococcus pneumoniae* produces pili that function as adherence factors to bind to epithelial cells in the human upper respiratory tract. In this study, we investigated the prevalence of pilus islets (PIs) in *S. pneumoniae* strains carried by healthy children below 5 years of age prior to pneumococcal vaccination in 2012 in Lombok Island, Indonesia. In all, 347 archived *S. pneumoniae* isolates were screened using polymerase chain reactions for the presence of *rrgC* and *pitB* genes representing pilus islet 1 (PI-1) and pilus islet 2 (PI-2), respectively. We found that 40 isolates (11.5 %) contained the PI genes: 5.2% carried both PI-1 and PI-2, and 3.5 and 2.9% carried PI-1 and PI-2, respectively. Furthermore, we found that most of the strains carrying either of the PIs belonged to the vaccine serotypes 19F and 19A and were less susceptible to chloramphenicol and tetracycline.

## Introduction

*Streptococcus pneumoniae* (the pneumococcus) expresses several virulence factors to promote its survival in the host, including pili, which are multimeric filamentous surfaces anchored to the peptidoglycan [1–3]. Pili are involved in host cell adhesion of pneumococci to epithelial cells to help the bacteria to escape from macrophage clearance, form a biofilm and be resistant to antibiotics [4–6]. Two types of pilus islets (PIs) have been reported in pneumococcus, pilus islet type 1 (PI-1) and pilus islet type 2 (PI-2) [2]. PI-1 is a 14 kb gene-encoding region of the islet and consists of seven genes (*rlrA*, *rrgA*, *rrgB*, *rrgC*, *srtB*, *srtC* and *srtD*), with RrgB as the major subunit that forms the backbone of the structure, and RrgA and RrgC as the ancillary proteins [7, 8]. PI-1 is commonly found in pneumococcal serotypes 4, 6B, 9V, 14 and 19F [9]. PI-2 is only a 6.6 kb gene-encoding region of the islet and consists of five genes (*pitA*, *sipA*, *pitB*, *srtG1* and *srtG2*), with PitB as the backbone subunit [7, 10]. PI-2 has been reported to be associated with serotypes 1, 2, 7F, 19A and 19F [9, 10].

Several studies have reported the prevalence of PI types in different regions. In Russia, it was reported that among 49 *S *. *pneumoniae* isolates of serotype 19A isolated from healthy children, 59 % (*n*=29) carried PI-1, whereas 14 % (*n*=7) carried PI-2 [11]. In Japan, among 151 isolates of *S. pneumoniae* isolated from patients suffering on pneumococcal disease, 34 isolates that carried PIs belonged to serotypes 19F, 23F, 19A, 6E, 15B and 35B [12]. Furthermore, a study from Portugal reported that in hospital inpatient care patient samples collected between 2008 and 2011 32 % of *S *. *pneumoniae* isolates carried pilus genes [13]. Hjálmarsdóttir *et al*. reported that genes encoding pilus, PI-1 and/or PI-2, in *S. pneumoniae* were carried by healthy Icelandic children and were mainly found in vaccine serotypes [7]. Recently, it was reported that the prevalence of *S. pneumoniae* carrying PI-1, PI-2 and both PI-1 and PI-2 was 16.6, 3.2 and 2 %, respectively, among carriage isolates from Brazilian children below 6 years of age [14]. At present, there are no studies reporting the prevalence of PIs in *S. pneumoniae* strains carried by the Indonesian population. Therefore, this retrospective study aimed to investigate the prevalence of PIs in *S. pneumoniae* isolated from healthy children below 5 years of age in Lombok Island, Indonesia.

## Methods

In all, 347 archived isolates of *S. pneumoniae* obtained from healthy children below 5 years of age (mean age was 25.7 months) in Central Lombok Regency, Lombok Island, Nusa Tenggara Barat Province, Indonesia were used in this study [15]. The pneumococcal strains were isolated from the nasopharyngeal swab specimens of these healthy children in 2012 prior to the administration of the pneumococcal vaccination. The isolates were subcultured onto a 5 % sheep blood agar plate and incubated at 37 °C in a 5 % CO_2_ atmosphere for 20 h. Bacterial DNA was extracted as described previously [16]. The polymerase chain reaction (PCR) targeting genes for PI-1 and PI-2 was performed as described previously [17]. Briefly, the reaction mixture comprising GoTaq Green Master Mix (Promega, Madison, WI, USA), the primers for PI-1: *rrgCmut* forward 5′-AACAGCCTGCTGGTTATGC-3′ and *rrgCmut* reverse 5′-TAGAGCGAACATAGTAAAGAC-3′ and the primers for PI-2: *pitBmut* forward 5′-GAGTGTCTGGGGAGAATTCCTTTAC-3′ and *pitBmut* reverse 5′-GGTTATTGCTGAATTAGGATCCGC-3′ at 10 µM concentration, 1.0 µl of DNA template and nuclease-free water was prepared to a final volume of 25 µl. The PCR condition was set as follows: 96 °C for 3 min, followed by 30 cycles of 94 °C for 10 s, 55 °C for 30 s and 72 °C for 30 s, with a final extension at 72 °C for 10 min [17]. Serotyping and antibiotic susceptibility testing were performed using the multiplex sequence PCR and disc diffusion methods, respectively, as described previously [16, 18]. For serotype determination, the primers used targeted 40 out of 93 known *S. pneumoniae* serotypes and the *cpsA* gene as an internal positive control [18]. In this study, we included different pneumococcal serotypes, i.e. 6A/6B (*n*=74), 19F (*n*=39), 23F (*n*=39), 15B/15C (*n*=21), 19A (*n*=14), 14 (*n*=13), 10A (*n*=10), 11A/11D (*n*=10), 35B (*n*=7), 34 (*n*=6), 15A/15F (*n*=6), 18 (*n*=6), 22F/22A (*n*=6), 3 (*n*=4), 35F/47F (*n*=4), 20 (*n*=3), 31 (*n*=3), 7F/7A (*n*=3), 1 (*n*=2), 4 (*n*=1), 38 (*n*=1), 17F (*n*=1) and nontypeable (NT, *n*=74) [15]. Antibiotic susceptibility testing was performed by the disc diffusion method according to the Clinical and Laboratory Standards Institute (CLSI) guidelines for chloramphenicol, clindamycin, erythromycin, trimethoprim/sulfamethoxazole, tetracycline and oxacillin discs [15].

## Results

### PCR analysis for detecting PI-1 and PI-2 genes in *S. pneumoniae* isolates and serotyping

**Fig. 1.**
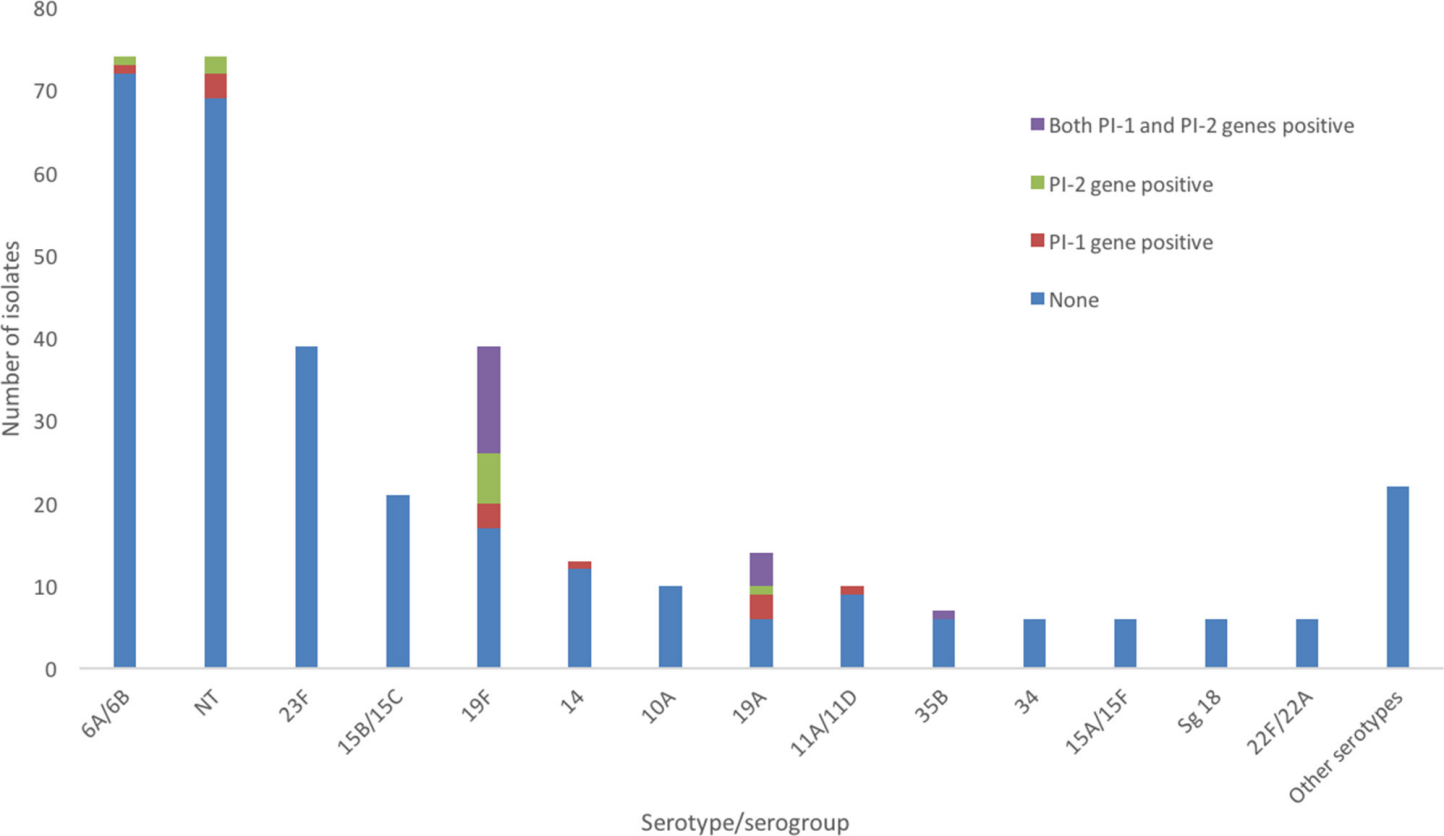
Presence of pilus islet genes according to pneumococcal serotypes/serogroups. NT, nontypeable.

We found that 11.5 % (40/347) of pneumococcal isolates contained PI genes. The prevalence of isolates carrying PI-1, PI-2, or both PI-1 and PI-2 was 3.5 % (12/347), 2.9 % (10/347) and 5.2 % (18/347), respectively. Pneumococcal serotypes 19A, 19F and NT were the predominant serotypes that carried PI-1 (three strains each), followed by 14, 11A/11D and 6A/6B (one strain each) (Table 1). Furthermore, we observed that pneumococcal serotype 19F was the most common serotype (6/10 strains) among isolates carrying PI-2 genes followed by NT (two strains), 19A and 6A/6B (one strain each). In addition, we also observed that among the *S pneumoniae* isolates carrying both PI-1 and PI-2 genes, serotype 19F was the major serotype (13/18 strains, 72 %) followed by 19A (4 strains, 22 %) and 35B (1 strain, 6 %). In general, we observed that serotype 19F was the predominant serotype (22/40 strains, 55 %,) carrying pilus genes, followed by 19A (8/40 strains, 20 %) and NT (5/40, 13 %) (Table 1, Fig. 1). In this study, we show that 94 % of isolates carrying pilus genes were vaccine serotypes, with 19F (63 %) being predominant, followed by 19A (23 %), 6A/6B (6 %) and 14 (3 %).

**Table 1.** Distribution of pilus islets in *Streptococcus pneumoniae* isolates from 347 healthy children below 5 years of age in Lombok Island, Indonesia

Pilus islet (PI)	No. of isolates (%)	Serotype/serogroup† (*n*)
PI-1	12 (3.5)	19F (3); 19A (3); NT* (3); 14 (1); 11A/11D (1); 6A/6B (1);
PI-2	10 (2.9)	19F (6); NT (2); 19A (1); 6A/6B (1);
PI-1+PI-2	18 (5.2)	19F (13); 19A (4); 35B (1)
Any PIs	40 (11.5)	19F (22); 19A (8); 6A/6B (2); 14 (1); 11A/11D (1); 35B (1); NT (5)

*nt, nontypeable.

†Serotype/serogroup data were obtained from the published data of Hadinegoro *et al*. [15].

By comparing serotypes, we found that serotypes 19F and 19A were most dominant in the isolates carrying PI genes [56 % (22/39) and 57 % (8/14), respectively], while the other serotypes remained under 15 % (Table 2). Other serotype groups (*n*=116) were found to be negative by PCR detection for either *rrgC* (PI-1) or *pitB* (PI-2) genes, including serotypes 23F (*n*=39), 15B/C (*n*=21) and 10A (*n*=10) (Table 2). Serotype 19A was predominant for the carriage of PI-1 genes (21 %, 3/14), while serotype 19F contained PI-2 genes (15 %, 6/39). Serotypes commonly found to contain both PI-1 and PI-2 genes were of serotype 19F (33 %, 13/39), followed by serotype 19A (29 %, 4/14) and serotype 35B (14 %, 1/7) (Table 2, Fig. 1).

**Table 2.** The proportion of pneumococcal serotypes carrying pilus islet genes

Serotype/ serogroup *	Isolates, *n*	Isolates carrying pilus islet genes, *n* (%)	PI-1 gene-positive, *n* (%)	PI-2 gene-positive, *n* (%)	Both PI-1 and PI-2 gene-positive, *n* (%)
19F	39	22 (56)	3 (8)	6 (15)	13 (33)
19A	14	8 (57)	3 (21)	1 (7)	4 (29)
14	13	1 (8)	1 (8)	0	0
6A/6B	74	2 (3)	1 (1)	1 (1)	0
11A/11D	10	1 (10)	1 (10)	0	0
35B	7	1 (14)	0	0	1 (14)
Others	116	0	0	0	0
nt†	74	5 (7)	3 (4)	2 (3)	0

*Serotyping was performed by multiplex PCR as previously reported by Hadinegoro *et al*. [15].

†nt, nontypeable.

### Antibiotic susceptibility testing of *S. pneumoniae* isolates

**Fig. 2.**
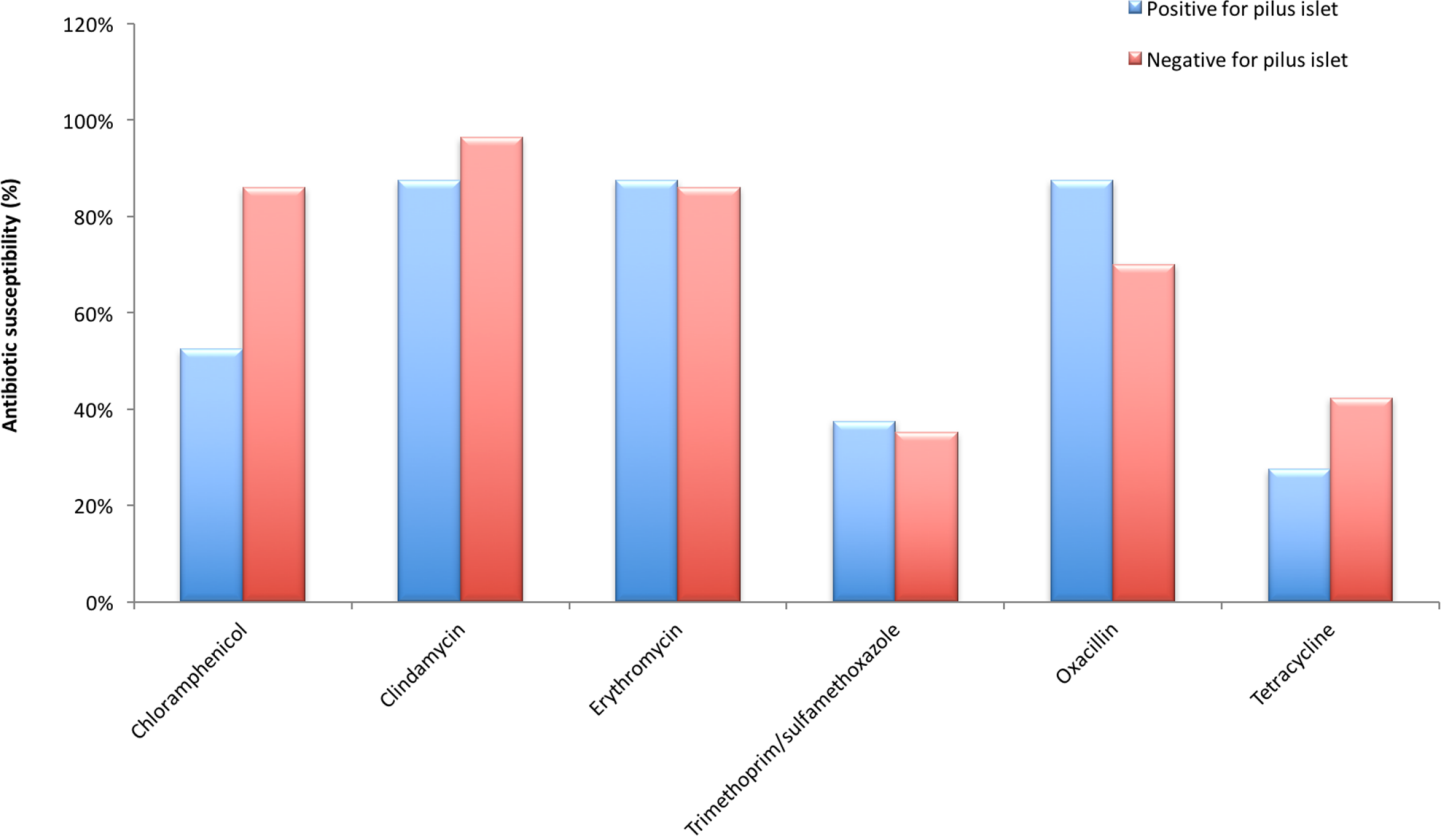
Antibiotic susceptibility profile among pneumococcal isolates carrying *(n*= 40) and not carrying (*n*=307) pilus islet genes.

In this study, we found that the strains carrying PIs were susceptible to erythromycin (88 %), clindamycin (88 %), oxacillin (88 %), chloramphenicol (53 %), trimethoprim/sulphamethoxazole (38 %) and tetracycline (28 %) (Fig. 2). Strains without any PIs were susceptible to erythromycin (86 %), clindamycin (96 %), oxacillin (70 %), chloramphenicol (86 %), trimethoprim/sulphamethoxazole (35 %) and tetracycline (42 %). Comparison among isolates carrying and not carrying PI genes showed that isolates with PIs were less susceptible to chloramphenicol (PI-positive=53 % vs PI-negative=86 %; Fisher’s exact test *P* <0.0001) and tetracycline (PI-positive=28 % vs PI-negative=42 %; Fisher’s exact test *P*=0.0871) (Fig. 2).

## Discussion

In this study, we aimed to evaluate the prevalence of PIs in *S. pneumoniae* strains isolated from healthy children below 5 years of age prior to pneumococcal vaccination in Indonesia. We found that the prevalence of strains carrying PI-1 was similar to that of strains carrying PI-2 [including those carrying both *rrgC* (PI-1) and *pitB* (PI-2) genes], i.e. PI-1=8.6 % (30/347) vs PI-2=8.1 % (28/347). However, Bagnoli *et al*. found that the prevalence of PI-1 is almost twice that of PI-2 (31.5 % for PI-1 vs 16.4 % for PI-2) among *S. pneumoniae* isolates where a majority had been isolated from clinical and invasive specimens from different countries [10]. Hjálmarsdóttir *et al*. reported that 33.7 % of pneumococcal strains isolated from healthy preschool children in Iceland where the great majority of the children had not received pneumococcal vaccines in 2009 showed the presence of PI-1, while only 9.5 % of strains showed the presence of PI-2 [7]. We suggested that the proportion of pneumococcal serotypes carrying the PI genes contributed to the prevalence of PIs in this study. We identified pneumococcal serotype 6A/6B as the major strain from Lombok Island, Indonesia (74/347, 21 %), but only two isolates carried PI genes (2/74, 3 %) (Fig. 1). Meanwhile, the study in Iceland showed that the PI-1 prevalence for serotypes 6B and 6A was 82.8 % (48/58) and 45.8% (22/48), respectively [7]. Another study at the Thailand–Myanmar border found that 77 % of the pneumococcal serotype 23F isolates (63/82) carried PI-1 [19]. However, we did not detect pneumococcal serotype 23F (0/39) for any PI genes from isolates from Lombok Island (Fig. 1).

Since the majority of the strains carrying PIs belonged to the serotypes 19F and 19A (75 %), we compared the antibiotic susceptibility profiles of serotypes 19F and 19A with or without PI genes. We found that serotypes 19F and 19A, which carried PIs genes, were less susceptible to chloramphenicol compared to those without PIs genes (43 vs 65%; Fisher’s exact test *P*=0.1661) (data not shown). However, it was reported that pneumococcal strains carrying both PI-1 and PI-2 are often related to penicillin-non-susceptible pneumococci and vaccine-type strains [7]. Furthermore, a study in Japan also reported that both PI-1 and PI-2 genes in serotype 19F and 19A lead to penicillin-non-susceptible pneumococci [12]. In addition, the serotypes 19F and 19A carrying both PIs genes are also found to be multidrug-resistant isolates [12]. Moreover, we also discovered that pneumococcal strains (serotypes 19F and 19A) carrying both PI-1 and PI-2 genes were less susceptible to chloramphenicol, trimethoprim/sulphamethoxazole and tetracycline. Our findings indicate that there is an association between serotypes, PI genes and antimicrobial non-susceptibility. These findings may also explain the fact that serotypes 19F and 19A are the most common serotypes colonizing the human nasopharynx in the Indonesian population, as they are supported by the pilus to strengthen their adherence to epithelial cells and are relatively resistant to antibiotic stress, leading to a higher survival rate during colonization. In conclusion, we showed that PIs are present in pneumococcal isolates carried by healthy children below 5 years of age in Lombok Island prior to pneumococcal vaccination in Indonesia. Furthermore, we found that most of the strains carrying PIs belong to the vaccine serotypes 19F and 19A and are less susceptible to chloramphenicol and tetracycline.
